# Investigating G protein signalling bias at the glucagon-like peptide-1 receptor in yeast

**DOI:** 10.1111/bph.12716

**Published:** 2014-07-17

**Authors:** C Weston, D Poyner, V Patel, S Dowell, G Ladds

**Affiliations:** 1 Division of Biomedical Cell Biology, Warwick Medical School, University of WarwickCoventry, UK; 2 Aston Pharmacy School, Aston UniversityBirmingham, UK; 3 Education and Development, Warwick Medical School, University of WarwickCoventry, UK; 4 Departments of Biological Reagent and Assay Development, GlaxoSmithKlineStevenage, UK

**Keywords:** GPCR, glucagon-like peptide-1 (GLP-1), glucagon, diabetes, signal bias, G proteins, liraglutide, exenatide

## Abstract

**BACKGROUND AND PURPOSE:**

The glucagon-like peptide 1 (GLP-1) receptor performs an important role in glycaemic control, stimulating the release of insulin. It is an attractive target for treating type 2 diabetes. Recently, several reports of adverse side effects following prolonged use of GLP-1 receptor therapies have emerged: most likely due to an incomplete understanding of signalling complexities.

**EXPERIMENTAL APPROACH:**

We describe the expression of the GLP-1 receptor in a panel of modified yeast strains that couple receptor activation to cell growth via single Gα/yeast chimeras. This assay enables the study of individual ligand–receptor G protein coupling preferences and the quantification of the effect of GLP-1 receptor ligands on G protein selectivity.

**KEY RESULTS:**

The GLP-1 receptor functionally coupled to the chimeras representing the human Gαs, Gαi and Gαq subunits. Calculation of the dissociation constant for a receptor antagonist, exendin-3 revealed no significant difference between the two systems. We obtained previously unobserved differences in G protein signalling bias for clinically relevant therapeutic agents, liraglutide and exenatide; the latter displaying significant bias for the Gαi pathway. We extended the use of the system to investigate small-molecule allosteric compounds and the closely related glucagon receptor.

**CONCLUSIONS AND IMPLICATIONS:**

These results provide a better understanding of the molecular events involved in GLP-1 receptor pleiotropic signalling and establish the yeast platform as a robust tool to screen for more selective, efficacious compounds acting at this important class of receptors in the future.

## Introduction

The observation that a greater insulin response occurs following oral glucose administration than with an equivalent i.v. challenge leads to the proposal of the incretin effect (McIntyre *et al*., 1964). Glucagon-like peptide-1 (GLP-1) is an incretin released from the intestine following nutrient ingestion. GLP-1 acts to regulate blood glucose primarily through the stimulation of insulin release from pancreatic β-cells (Baggio and Drucker, 2007). This insulinotropic action of GLP-1 is mediated via activation of a classical seven transmembrane, GPCR, the GLP-1 receptor (see Alexander *et al*., 2013). Due to its central role in glucose-induced insulin release, the GLP-1 receptor has become a major focus for therapeutic strategies to target type 2 (insulin-insensitive) diabetes (Nauck, 2011).

In addition to insulin insensitivity, type 2 diabetic patients display lowered GLP-1 concentrations and a reduced ability to promote insulin secretion (Quddusi *et al*., 2003). Administration of exogenous GLP-1 peptide potentiates glucose-dependent insulin secretion and, importantly, regulates the hyperglycaemia observed in these patients (Holst and Seino, 2009). However, the active form of GLP-1 [GLP-1 (7-36) amide] has a very short plasma half-life of a few minutes, being rapidly cleaved by the serine protease dipeptidyl peptidase-IV (DPP-IV) to an inactive form [GLP-1 (9-36) amide] (Mentlein *et al*., 1993). Several, DPP-IV-insensitive peptide analogues to GLP-1, which act as GLP-1 receptor agonists, have been developed to demonstrate improved plasma half-life. These so-called mimetics, exenatide and liraglutide have been approved for clinical use in the treatment of type 2 diabetes (Scott *et al*., 2013).

Despite the undoubted advantages that these mimetics provide for immediate weight loss and in managing previously uncontrolled type 2 diabetes, a number of concerns remain regarding their long-term use including an increased risk of pancreatitis and cancer (Butler *et al*., 2013). Significantly, despite having a high degree of sequence homology, the different ligands display markedly different clinical effects (Pabreja *et al*., 2014) leading to speculation of multiple GLP-1 receptor isoforms. However, these results could also be explained by a combination of ligand-directed signalling bias and the ability of the GLP-1 receptor to couple to multiple G proteins. Combined, these observations highlight an incomplete understanding of the signalling pathways underlying the GLP-1 receptor response. These concerns lead to the classification of the mimetics in ‘tier 2’ of the consensus algorithm for initiation and adjustment therapy for the American Diabetes Association and European Association for the Study of Diabetes. This category is for ‘less well-validated’ treatments and should therefore be used in patients as a last line (Nathan *et al*., 2009). A better understanding of the signalling responses modulated by the GLP-1 receptor is therefore required to enable the more efficient design and use of these therapies.

Most traditional assays used to date to investigate GLP-1 receptor activation can be influenced by crosstalk of the different signalling pathways. Here we report the use of a simple, robust, *Saccharomyces cerevisiae* system in which single GPCR-G protein couplings can be observed (Dowell and Brown, 2002) to study the effect of G protein subunit on GLP-1 receptor activation. Previous studies have highlighted the robust nature of yeast signalling assays for determining relative efficacies of agonists and affinity values for both agonists and antagonists (Stewart *et al*., 2009). Further, the individual nature of the GPCR-G protein coupling permits identification of potential signalling bias in the absence of competing G proteins and other GPCRs.

Using the yeast signalling assays, here we describe, for the first time, the G protein bias profiles of the clinically used GLP-1 mimetics. In addition, we have investigated the ability of two small-molecule allosteric modulators; compound 2 (6,7-dichloro-2-methylsulfonyl-3-N-tert-butylaminoquinoxaline, Knudsen *et al*., 2007) and BETP [4-(3-benzyloxyphenyl)-2-ethylsulfinyl-6-(trifluoromethyl) pyrimidine] (Sloop *et al*., 2010) to modulate GLP-1 receptor signalling. Our data provide direct evidence for multiple high affinity couplings for all GLP-1 receptor agonists and establishes the yeast platform as a robust tool to screen for more selective highly efficacious mimetic compounds in the future.

## Methods

### Constructs and DNA manipulation

cDNA constructs of the human GLP-1 receptor containing a myc-tag was donated by Professor Patrick Sexton (Monash University, Australia). cDNA for human GLP-1 receptors was provided by Dr Graeme Wilkinson (AstraZenica, UK). cDNA for the glucagon receptor (GCGR) was purchased from the Missouri University of Science and Technology cDNA Resource Centre (http://cdna.org). DNA manipulations were performed using standard methods. Oligonucleotides were supplied by Invitrogen. PCR amplification used FastStart Taq polymerase (Roche Diagnostics, Burgess Hill, UK). All constructs were sequenced by GATC (GATC Biotech, London, UK) prior to use.

### Yeast growth media and transformations

General yeast procedures were performed as described previously (Dowell and Brown, 2002). Culture media used was rich yeast peptone dextrose adenosine (YPDA) (for routine cell growth) or synthetic dropout (SD) media lacking uracil or histidine as appropriate. Yeast transformations were achieved by using the lithium acetate/single-stranded DNA/polyethylene glycol method as previously described (Gietz and Schiestl, 2007).

### Yeast strain construction

Production of *S. cerevisiae* dual reporter strains expressing chimeras of five C-terminal amino acids of human Gα protein with the yeast GPA1, 1-467, (GPA1/Gα) has been described previously (Brown *et al*., 2003). Mammalian GPCRs were introduced into the yeast strains (MMY14, MMY24, MMY25 and MMY28) by cloning an expression cassette consisting of the *GAPDH* promoter, the GPCR of choice and the *CYC1* terminator sequence into pRS306 and integrated at the *ura3* locus. Intergrants were selected upon their ability to generate a β-galactosidase activity response above basal when stimulated with 10 μM GLP-1 (7-36) amide or glucagon as appropriate for the GPCR being studied. β-galactosidase activity was measured as described previously (Dohlman *et al*., 1995; Brown *et al*., 2000) using o-nitrophenyl-β-D-galactopyranoside as a substrate. For chimeric strains that did not initially appear to functionally couple (*n* = 16 isolates) to GLP-1 receptors, expression of the receptor was confirmed using immunoblotting.

### Yeast growth assay

GPCR activation was measured using an assay for yeast growth adapted from Bertheleme *et al*. (2013). The strains used contain the *HIS3* gene under the control of the pheromone-responsive *FUS3* promoter. Receptor agonism induces expression of the reporter gene enabling growth in media lacking histidine (-HIS). Cell growth was determined using FDGlu as a substrate, which is converted to fluorescein by exoglucanase, an enzyme secreted by dividing yeast cells (Dowell and Brown, 2009). Initially, cells were grown for 24 h in SD media lacking uracil (-URA) at 30°C to select for only those expressing the receptor. Then, in a change to other documented examples of GPCRs expressed in these reporter strains, cells were cultured, overnight at 30°C (following a 1 in 10 dilution) in SD-URA-HIS media to remove basal activity and provide a larger signalling range to be detected upon stimulation with agonists. Finally, the cultures were diluted in SD-URA-HIS media, supplemented with FDGlu at a final concentration of 20 μM, to an OD_600_ of 0.02. Fluorescein signal was detected as an increase in fluorescence (excitation wavelength = 485 nm, emission wavelength = 535 nm) as a measure of growth. Different concentrations of ligands (0.01 nM–100 mM) were added and yeast growth was measured using either a TECAN Infinite M200 microplate reader (TECAN Ultra Evolution, Reading, UK) or a Mithras LB940 microplate reader (Berthold Technologies, Harpenden, UK) for 20 h.

### Membrane preparations and immunoblotting

Cultures, 200 mL, of yeast cells were grown in YPDA and plasma membrane extracts prepared as described previously (Ladds and Davey, 2000; Ladds *et al*., 2005b). Samples were collected in 50 μL resuspension buffer (20% sucrose, 10 mM Tris-HCl pH 6.8) and diluted 1:1 with 2× Laemmli sample buffer containing 6 M urea (Ladds and Davey, 2000). Extracts were heated to 37°C for 10 min before being subjected to SDS-PAGE using the Nu-PAGE technology (Invitrogen). Following separation using SDS-PAGE, samples were transferred to PVDF membrane using a semi-dry blotter. Western blotting was performed using a GLP-1 receptor polyclonal antibody (Abcam, Cambridge, UK) and a donkey anti-rabbit IgG HRP conjugate (Amersham, International, Little Chalfont, UK) as the secondary antibody. HRP activity was detected using enhanced chemiluminescence reagents EZ-ECL (Geneflow, Lichfield, UK) and visualized using a Syngene G:Box gel documentation system (Syngene, Cambridge, UK) as described in Bond *et al*. (2013).

### Cell culture and transfections

HEK293T cells were provide by Dr Jügen Müller (University of Warwick) and cultured in DMEM (Invitrogen) supplemented with 10% FCS and L-glutamine in a humidified 5% CO_2_–95% air incubator at 37°C. Cells were transfected with Fugene 6 (Roche, Indianapolis, IN, USA) in accordance with the manufacturer's instructions. Transfected cell lines were grown for 48 h enabling maximal expression before assaying.

### cAMP accumulation assays

Growth medium was removed from the cells and replaced with serum free DMEM containing 500 mM IBMX for 30 min. Peptides in the range 10 pM to 1 mM were added for a further 15 min. Ice-cold ethanol (95–100% v v^−1^) was used to extract cAMP, which was subsequently measured by radioreceptor assay as previously described (Poyner *et al*., 2014).

### Fluorescent microscopy of yeast

To visualize GLP-1 receptor expression in yeast cells, a C-terminal in-frame fusion construct between the GLP-1 receptor and 3xmCherry ORFs fused together (separated by a Asp-Gly linker) was generated using a two-step cloning technique as described previously (Ladds *et al*., 2005b). To generate an expression cassette, GLP-1 receptor-3xmCherry was cloned into pRS306 containing the *GAPDH* promoter and the *CYC1* terminator sequence separated by a B*am*HI restriction site. This was integrated at the *ura3* locus. Positive transformants were grown in YPDA to a density of 5 × 10^6^ cells mL^−1^. Then 100 μL of culture was harvested and the cells washed in growth media. Cell suspension (3 μL) was transferred to slides (Sigma) and viewed using a Personal DeltaVision system (Applied Precision, Issaquah, WA, USA) equipped with a Photometric CoolSNAP HQ camera (Roper Scientific, Trenton, NJ, USA). Deconvolution was applied to images for visual clarity.

### Data analysis

Data were analysed using Prism 6.0c (Graphpad Software, San Diego, CA, USA). Concentration-response curves were fitted using the three-parameter logistic equation to obtain EC_50_ and E_max_ values. The operational model for partial agonism (Black and Leff, 1983) was used to obtain values of efficacy (log τ) and the equilibrium dissociation constant (log *K*_A_). These values were then used to quantify signalling bias as change in log (τ/*K*_A_) relative to the natural GLP-1 receptor ligand, GLP-1 (7-36) amide (Figueroa *et al*., 2009). Statistical differences were analysed using one-way anova with Bonferroni's or Dunnett's multiple comparisons or Student's test as appropriate and a probability (*P*) < 0.05 was considered significant.

### Materials

GLP-1 (7-36) amide, oxytomodulin, glucagon, exendin-4 (exenatide) and exendin-3 were synthesized by Alta Biosciences (University of Birmingham, Birmingham, UK) and prepared as 1 mM stocks in water. Liraglutide was supplied by George Eliot Hospital NHS Trust (Nuneaton, UK). Small-molecule agonists, compound 2 and BETP were obtained from Sigma-Aldrich (St Louis, MO, USA) and prepared as 100 mM stocks in DMSO. Yeast nitrogen base and yeast extract were purchased from Difco (Franklin Lakes, NJ, USA). Flurorescein-Di-β-D-glucopyranoside (FDGlu) was purchased from Invitrogen (Paisley, UK). All other reagents were purchased from Sigma-Aldrich.

## Results

### The GLP-1 receptor can activate the yeast-mating pathway via specific G protein chimeras

Modification of the *S. cerevisiae* mating pathway to enable functional coupling of mammalian GPCRs to the pheromone response pathway has enabled the generation of a genetically tractable system in which to express human receptors to examine their signalling properties (Brown *et al*., 2000; Stewart *et al*., 2009). These strains are deleted for the pheromone receptor, to provide a null background and contain a dual reporter (*FUS1-lacZ* and *FUS1-HIS3*) gene under the control of the endogenous yeast pheromone response to give a quantitative assay for GPCR activation (Dowell and Brown, 2002).

**Figure 1:**
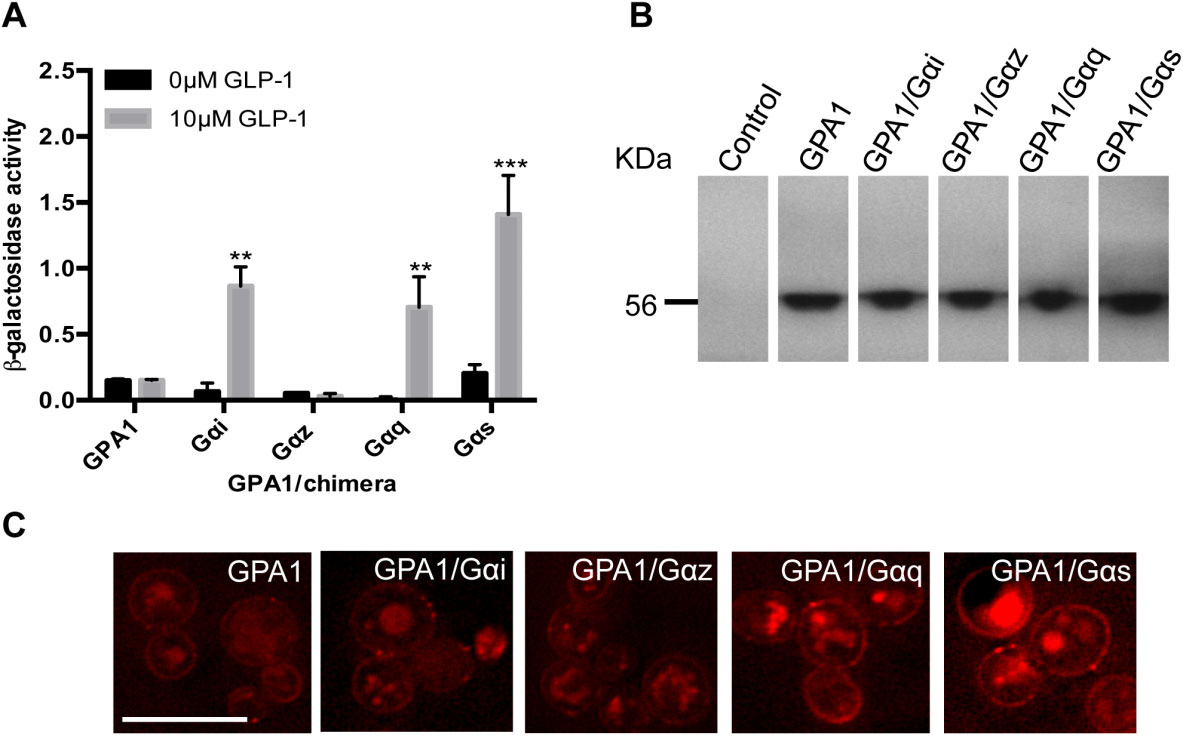
Activation of the yeast-mating pathway by the GLP-1 receptor. (A) Strains containing the GLP-1 receptor were stimulated with 0 or 10 μM GLP-1 for 20 h and assayed for activation of the *FUS1* > *lacZ* reporter gene. Data are mean of five independent experiments ± SEM. Data were determined as significantly different from the non-peptide response using Student's *t*-test where **P < 0.01, ***P < 0.001. (B) Expression of the GLP-1 receptor in yeast strains containing various GPA1/Gα chimeras was confirmed using immunoblotting. (C) A C-terminal 3xmCherry tag was engineered onto the GLP-1 receptor and expression at the plasma membrane was confirmed using fluorescence microscopy. Scale bar = 10 μm.

The GLP-1 receptor, under the control of a strong yeast promoter, was integrated into a panel of strains engineered to contain chimeric Gα-subunits in which the 5 C-terminal amino acids of GPA1 have been replaced with those of mammalian Gαs, Gαi, Gαz and Gαq. To determine which Gα-subunits the GLP-1 receptor signals through, yeast strains expressing the receptor were incubated with 10 μM of the potent, natural peptide ligand, GLP-1 (7-36) amide (hereafter referred to as GLP-1 for simplicity). An increase in reporter gene activity was observed when the G protein chimeras corresponding to Gαs, Gαi and Gαq were present, confirming that the receptor is functionally expressed in the yeast system and can couple to the pheromone response pathway (Figure 1A). Signalling was not observed via the un-modified GPA1 or Gαz subunits (*n* ≥ 16 isolates were screened for functionality). To ensure uniform expression across all yeast strains, cells were grown to mid log phase and cell extracts obtained before immunobloting using a GLP-1 receptor antibody (Figure 1B). Finally, efficient trafficking of the GLP-1 receptor was confirmed using a modified receptor engineered to contain 3mCherry fluorophores at the C-terminus (Figure 1C). Addition of the fluorophore to the C-terminus of the GLP-1 receptor did not alter the coupling profile of the receptor but confirmed plasma membrane localization in all strains.

**Figure 2:**
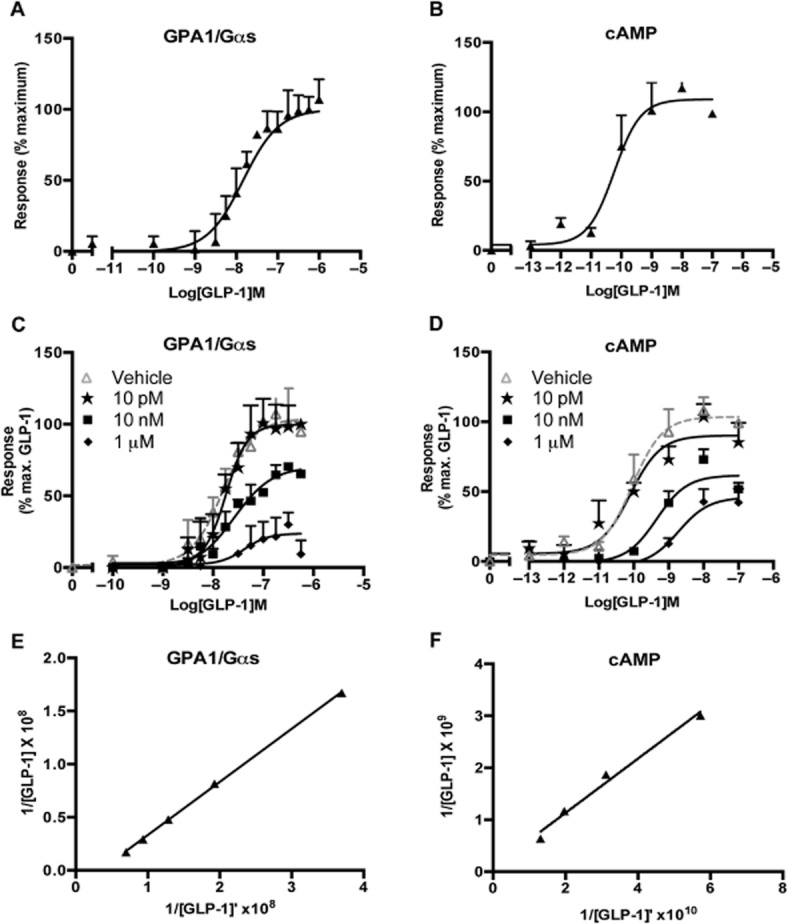
The GPA1/Gαs responses reproduce cAMP data for GLP-1 receptor agonism. (A) Dose-response curves to the natural GLP-1 receptor agonist, GLP-1, were constructed in the yeast strain containing the GPA1/Gαs chimera. Activation of the reporter gene was calculated as a percentage of the maximum response observed. (B) cAMP accumulation was determined following 30 min stimulation of transiently transfected HEK293T cells and expressed as a percentage of the maximum observed. (C) *S. cerevisiae* containing the GPA1/Gαs chimera and (D) transiently transfected HEK293T cells were stimulated with GLP-1 in the presence of the indicated concentrations of exendin-3. All data are expressed as a percentage of the maximal response in the absence of inhibitor and are mean of 5–8 independent experiments ± SEM. (E) and (F) Double reciprocal plots for GLP-1 in the presence (Y axis) and absence (X axis) of 10 nM exendin-3.

GPCR specificity for individual yeast chimeras has previously been demonstrated to generally conform to that observed in mammalian cells, indeed the GLP-1 receptor has been reported to couple through Gαs, Gαi and Gαq in other systems (Baggio and Drucker, 2007). We next compared the pharmacology of the GLP-1 receptor with respect to GPA1/Gαs activation in yeast cells and accumulation of cAMP in transiently transfected HEK293T cells. Initially, concentration-response curves were constructed to the GLP-1 receptor agonist, GLP-1, in both yeast and mammalian cells expressing the receptor (Figure 2A and B). Sigmoidal dose-response curves were observed allowing the calculation of an EC_50_ value for GLP-1 (10 nM and 150 pM, yeast and mammalian assays respectively). Notably, the agonist was more potent for the accumulation of cAMP than in the yeast system; however, both responses were robust over five repeats with minimal error (Table 1).

**Table 1:** Potency (pEC_50_) and maximal agonist response (E_max_) in the presence of the GLP-1 receptor antagonist, exendin-3

[Exendin-3] (M)	cAMP		GPA1/Gαs		GPA1/Gαi	
	pEC_50_^a^	E_max_^b^	pEC_50_^a^	E_max_^b^	pEC_50_^a^	E_max_^b^
Vehicle	10.2 ± 0.1***	100 ± 4.3***	7.9 ± 0.1	100 ± 9.0***	7.3 ± 0.07*	100 ± 9.2***
1 ± 10^−11^	10.1 ± 0.1***	100 ± 5.7***	7.8 ± 0.1	100 ± 9.2***	6.9 ± 0.1*	100 ± 8.1***
1 ± 10^−8^	9.2 ± 0.1***	61.7 ± 6.2***	7.6 ± 0.2	68 ± 7.1***	6.8 ± 0.2*	81 ± 7.1***
1 ± 10^−6^	8.9 ± 0.1***	49.5 ± 7.1***	7.3 ± 0.4	23 ± 7.0***	6.7 ± 0.1*	42 ± 5.9***
	cAMP		GPA1/Gαs		GPA1/Gαi	
	Slope^c^	p*K*_B_^d^	Slope^c^	p*K*_B_^d^	Slope^c^	p*K*_B_^d^
1 ± 10^−8^	0.5 ± 0.005	7.9 ± 0.1	0.5 ± 0.006	7.8 ± 0.1	0.13 ± 0.006	7.9 ± 0.1***

Values generated through fitting of a three-parameter logistic equation and represent the mean ± SEM from five independent experimental repeats.

a The negative logarithm of the agonist concentration required to generate half the maximal response.

b The maximal response to the ligand expressed as a percentage of that obtained in the absence of antagonist.

c Slope of linear regression from double reciprocal plot of GLP-1 in the presence and absence of 10 nM exendin-3.

d The negative logarithm of the equilibrium disassociation constant for the antagonist calculated using the method of Gaddum *et al*., 1955. Statistical significance compared with vehicle (*P < 0.05, **P < 0.01; ***P < 0.001) was determined by one-way anova with Dunnett's post-test.

To ensure faithful transfer of GLP-1 receptor pharmacology between mammalian cells and the yeast system, interaction experiments were performed using the antagonist exendin-3 (Göke *et al*., 1993). Increasing concentrations of exendin-3 inhibited the response to GLP-1 in both systems causing a dose-dependent rightward shift in the GLP-1 response and substantial depression of E_max_ (Figure 2C and D). Double reciprocal plots of equiactive concentrations of GLP-1 in the presence and absence of exendin-3 at 10 nM (Figure 2E and F) were constructed to calculate the dissociation constant for the receptor-antagonist complex (*K*_B_) (Gaddum *et al*., 1955). These values did not differ significantly between the two assay systems (Table 1). Despite the reduction in ligand potency, these data demonstrate that the pharmacology of the GLP-1 receptor in the yeast system broadly replicates that of mammalian cells, thereby establishing a useful platform for the investigation of G protein coupling in isolation from crosstalk between pathways.

### Using the yeast system to investigate competing GLP-1 receptor G protein pathways

Significantly, we identified in Figure 1 several functional GLP-1 receptor-G protein couplings that in mammalian cells act to regulate second messengers that can be assayed to determine receptor pharmacology. Of particular note, the primary physiological role of the GLP-1 receptor is to stimulate insulin secretion through the regulation of intracellular cAMP concentrations (Seino, 2012). GPCR-mediated control of cAMP levels occurs via regulation of adenylate cyclase activity, both positively (via Gαs) and negatively (via Gαi). The potential competing nature of the GLP-1 receptor couplings identified (Figure 1) highlight difficulties in the ability to use traditional mammalian assays to differentiate the proportion of a response attributable to each pathway. The effect of these two G proteins on GLP-1 receptor pharmacology was therefore focused upon for the remainder of this study.

**Figure 3:**
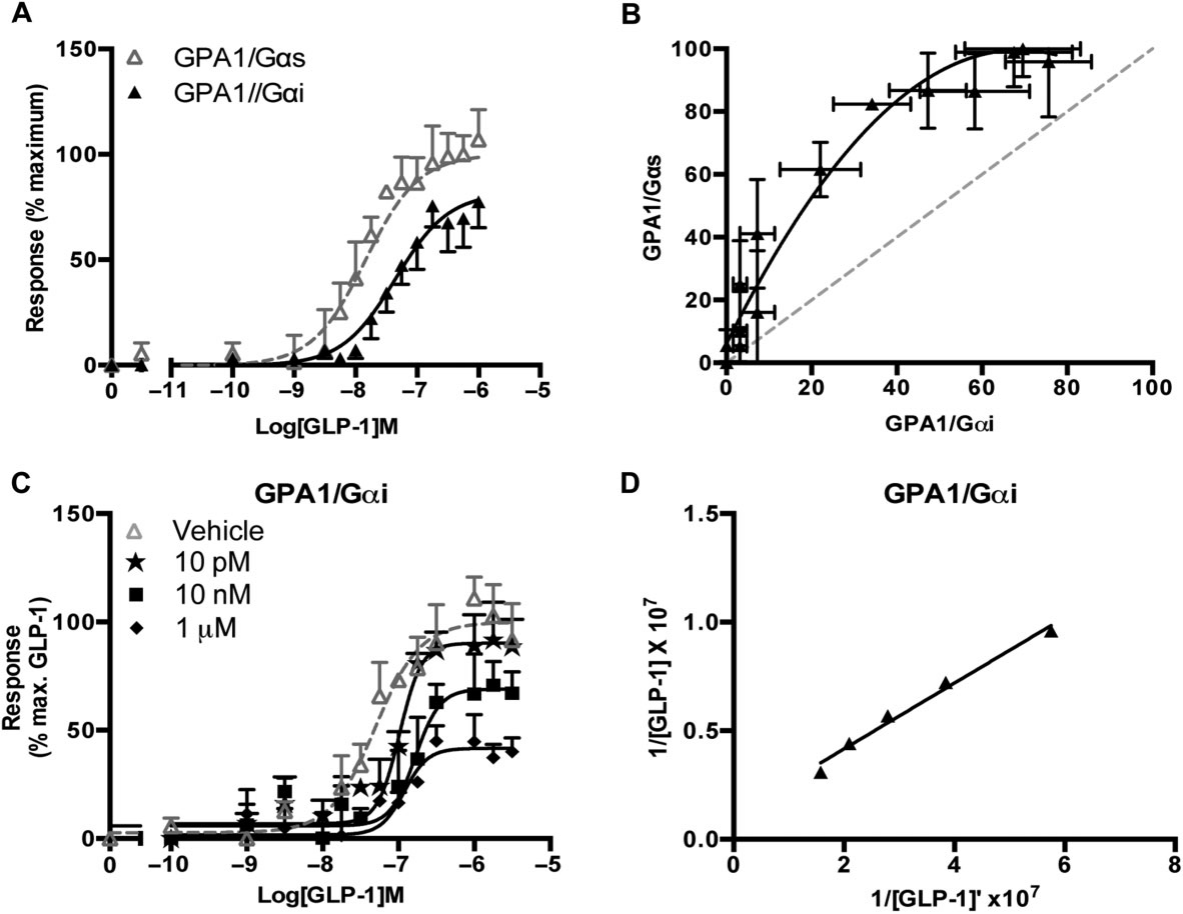
The G protein chimera influences the activity profile of GLP-1 receptor agonist. (A) Dose-response curves to the natural GLP-1 receptor agonist, GLP-1 were constructed in strains containing the GPA1/Gαs or GPA1/Gαi chimera. Activation of the reporter gene was calculated as a percentage of the maximum response observed in the GPA1/Gαs strain. (B) Equimolar comparison of bias, the normalized response at equivalent concentrations of agonist for each G protein chimera shown in (A) was plotted to allow visualization of any pathway preference. Dashed line represents no bias. (C) GPA1/Gαi chimera strain was stimulated with GLP-1 in the presence of the indicated concentrations of exendin-3. Reporter gene activity was determined following 20 h stimulation. All data are expressed as a percentage of the maximal response in the absence of inhibitor and are mean of 5–8 independent experiments ± SEM. (D) Double reciprocal plot for GLP-1 in the presence (Y axis) and absence (X axis) of 10 nM exendin-3.

To compare the effect of the G protein subtype on receptor activation, concentration-response curves were constructed to the GLP-1 receptor agonist, GLP-1 in *S. cerevisiae* strains containing either the Gαs or Gαi GPA1 chimeras (Figure 3A). The agonist was more potent when the receptor was coupled to GPA1/Gαs as could be seen through inspection of the values in Table 1. There was also a significant (*P* < 0.01, *n* = 5) reduction in the maximal response when the receptor coupled to the inhibitory G protein chimera.

An equimolar bias plot was generated (Rajagopal *et al*., 2003) to demonstrate the influence of the G protein subunit on ligand activity (Figure 3B). This graph enabled potential signalling preferences of ligands for individual pathways to be identified easily. The normalized responses for the different G protein chimeras at each concentration of GLP-1 were replotted against each other highlighting the preference of the receptor for GPA1/Gαs activation when stimulated with GLP-1.

To further investigate the signalling properties of the GLP-1 receptor in the chimeric yeast strain containing the GPA1/Gαi subunit, interaction experiments were performed using the antagonist, exendin-3. Similar to the observations in the GPA1/Gαs strain (Figure 2C), incubation with increasing concentrations of the antagonist resulted in a rightward shift of the GLP-1 dose-response curves (Figure 3C) decreasing the potency and maximal responses demonstrating an insurmountable effect. The dissociation constant for the antagonist, calculated from a double reciprocal plot (Figure 3D), did not differ significantly from that calculated in the presence of the GPA1/Gαs subunit (Table 1) suggesting that antagonism of the GLP-1 response by exendin-3 is independent of the G protein subunit.

### Investigating ligand bias of endogenous GLP-1 receptor agonists

**Figure 4:**
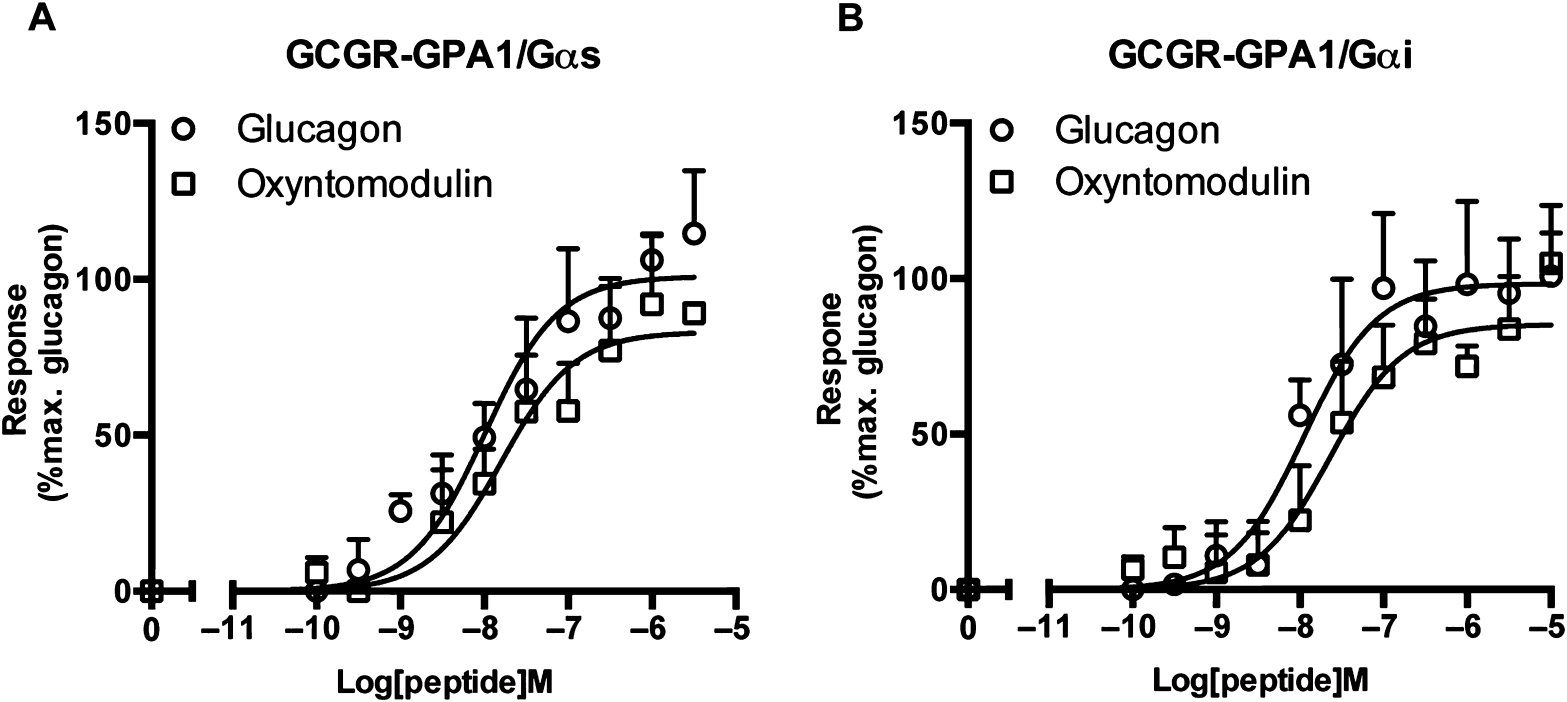
Functional coupling of the glucagon receptor to GPA1/Gαs and GPA1/Gαi. Dose-response curves to glucagon and oxyntomodulin at the glucagon receptor were constructed in yeast strains containing (A), the GPA1/Gαs and (B), the GPA1/Gαi chimera. Activation of the reporter gene was calculated as a percentage of the maximum response observed.

In addition to GLP-1, tissue-specific, post-translational processing of pro-glucagon produces both glucagon and the highly similar, oxyntomodulin (Schepp *et al*., 1996). Glucagon acts in opposition to insulin to promote glycogen breakdown thereby increasing blood glucose. Despite this, both glucagon and oxyntomodulin have been demonstrated to act via the glucagon receptor and the GLP-1 receptor to affect intracellular concentrations of cAMP (via Gαs and Gαi). To confirm that oxyntomodulin and glucagon were functional at receptors expressed in the yeast system, we first tested them at the glucagon receptor. Both glucagon and oxyntomodulin activated the glucagon receptor in a yeast strain containing either the GPA1/Gαs or GPA1/Gαi chimeras with similar potencies (Figure 4A and B, Table 2). Significantly, however, it is thought that oxyntomodulin, differing from glucagon only through an eight-amino acid C-terminal extension, exerts its affects, in mammalian cells primarily via the GLP-1 receptor, acting to reduce food intake in humans (Wynne *et al*., 2005; Schepp *et al*., 1996).

**Table 2:** Potency (pEC_50_) and maximal response (E_max_) values for peptide agonists of the glucagon receptor in yeast assays

Ligand	GPA1/Gαs		GPA1/Gαi	
	pEC_50_^a^	E_max_^b^	pEC_50_^a^	E_max_^b^
Glucagon	8.0 ± 0.1	101 ± 6.1	8.0 ± 0.3	98 ± 8.1
Oxyntomodulin	7.8 ± 0.2	85 ± 10.4	7.6 ± 0.2	85 ± 6.8

Values generated through fitting of a three-parameter logistic equation and represent the mean ± SEM from at least five independent experimental repeats.

a The negative logarithm of the agonist concentration required to generate half the maximal response.

b The maximal response to the ligand expressed as a percentage of that obtained in the absence of antagonist.

**Figure 5:**
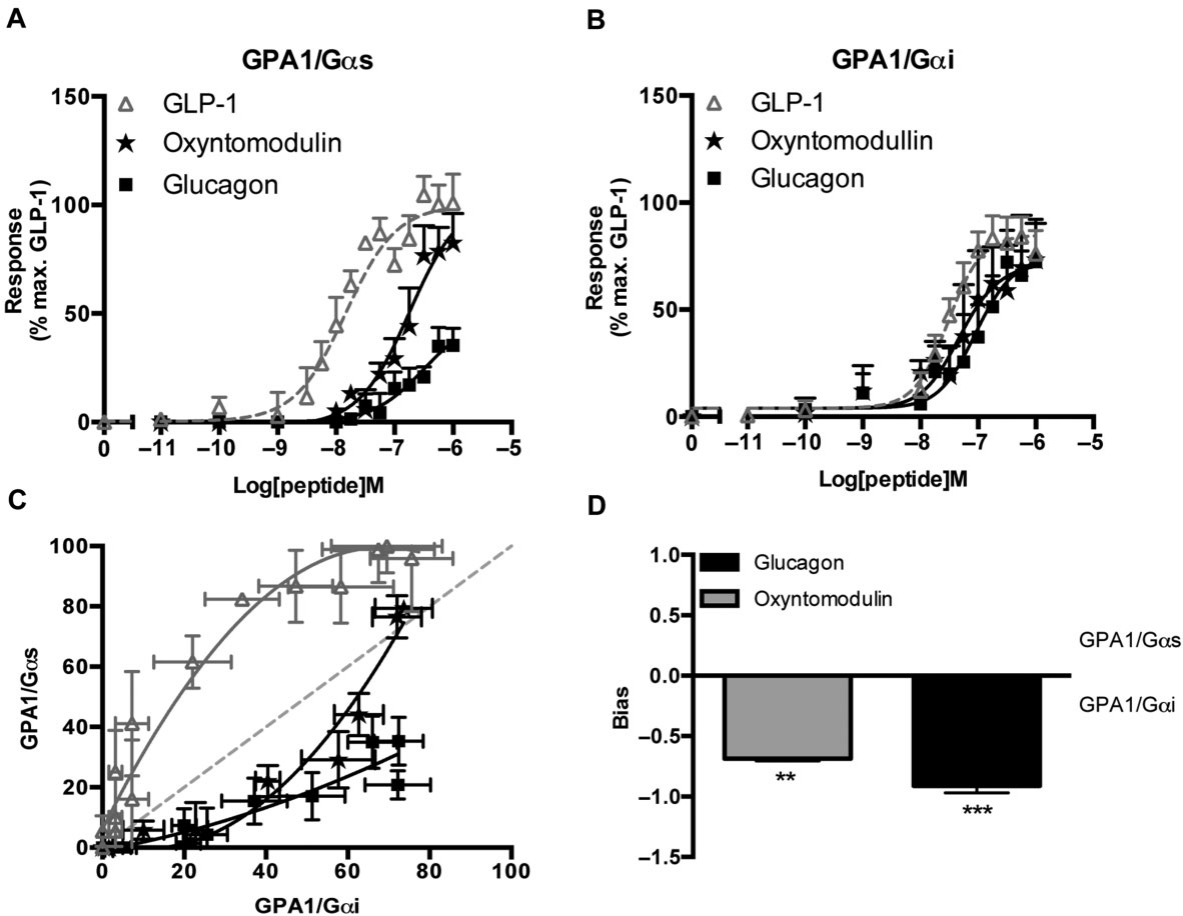
Activation of the GLP-1 receptor by glucagon and oxyntomodulin. (A) Yeast strains expressing the GLP-1 receptor were stimulated with GLP-1, glucagon or oxyntomodulin for 20 h and reporter gene activity determined following coupling of the receptor to (A) the GPA1/Gαs or (B) the GPA1/Gαi chimera. Data are expressed as a % of the maximum response achieved by GLP-1 in the GPA1/Gαs strain. (C) The normalized responses for equivalent concentrations of each ligand were plotted to observe the relative G protein bias where the dashed line represents no bias. (D) Signalling bias was calculated relative to GLP-1 as the change in log(τ/*K*_A_) ratio for the data in (A) and (B). Data were determined as statistically different (**P < 0.01, ***P < 0.001) from GLP-1, using a one-way anova with Bonferroni's post-test. All data are mean of 5–8 independent experiments ± SEM.

To further investigate the pharmacology of these ligands at the GLP-1 receptor in isolation from competing pathways, we next studied the ability of glucagon and oxyntomodulin to activate the yeast-mating response via the GPA1/Gαs and GPA1/Gαi chimeras. Both glucagon and oxyntomodulin caused a dose-dependent activation of the reporter gene (Figure 5A and B) with reduced potency compared with GLP-1 (Table 3). Both peptides displayed different signalling profiles relative to GLP-1 depending upon the G protein chimera present. Glucagon appears to be a weak (EC_50_ = 300 nM) agonist with respect to GPA1/Gαs activation possibly displaying only partial agonism with a significantly (*P* < 0.01, *n* = 5) reduced E_max_ (48.7 ± 6.6). However, a full, more potent (EC_50_ = 102 nM) response in the presence of GPA1/Gαi was observed (Table 3). Both oxyntomodulin and glucagon demonstrate signalling bias for the activation of the GPA1/Gαi chimera (Figure 5C). Differences in the log(τ/*K*_A_) ratios relative to GLP-1 indicated that both ligands display a significant (*P* < 0.01, *n* = 5) bias towards the inhibitory G protein (Figure 5D). These data provide an insight into the molecular mechanisms affected by GLP-1 receptor agonism and a possible explanation for the reduced potency of the glucagon ligands observed in mammalian cAMP assays (Jorgensen *et al*., 2007).

**Table 3:** Potency (pEC_50_), affinity (p*K*_A_) and coupling efficacy (log τ) values for various GLP-1 receptor agonists from yeast assays

Ligand	GPA1/Gαs		GPA1/Gαi		GPA1/Gαs		GPA1/Gαi	
	pEC_50_^a^	E_max_^b^	pEC_50_^a^	E_max_^b^	p*K*_A_^c^	log τ^d^	p*K*_A_^c^	log τ^d^
GLP-1	8.1 ± 0.10***	100 ± 5.4***	7.5 ± 0.1	75.7 ± 5.6	6.7 ± 0.2**	1.0 ± 0.2**	6.7 ± 0.3	0.7 ± 0.2
Glucagon	6.5 ± 0.40***	48.7 ± 6.6***	6.9 ± 0.5	82.6 ± 8.0	6.2 ± 0.2**	−0.1 ± 0.3**	6.4 ± 0.3	0.4 ± 0.2
Oxyntomodulin	6.7 ± 0.20***	103 ± 7.2***	7.7 ± 0.3	76.6 ± 7.0	5.4 ± 0.2**	1.3 ± 0.01**	6.8 ± 0.4	0.3 ± 0.1
Exenatide	6.5 ± 0.07***	53.6 ± 8.0***	6.6 ± 0.2*	87.7 ± 5.4	6.3 ± 0.4**	−0.02 ± 0.3**	6.2 ± 0.6	0.5 ± 0.4
Liraglutide	7.5 ± 0.06***	99.1 ± 7.9***	7.1 ± 0.06	84.6 ± 5.3	6.4 ± 0.4**	0.9 ± 0.3**	6.5 ± 0.7	0.5 ± 0.5

All values are mean ± SEM of five independent experimental repeats. Statistical significance compared with GLP-1 (*P < 0.05; **P < 0.01; ***P < 0.001) was determined by one-way anova with Dunnett's post-test.

a The negative logarithm of the agonist concentration required to produce a half-maximal response.

b The maximal response to the ligand expressed as a percentage of that obtained in the absence of antagonist.

c The negative logarithm of the equilibrium disassociation constant for each ligand generated through use of the operational model of agonism (Black and Leff, 1983).

d τ is the coupling efficiency parameter, generated by comparison to the natural ligand, GLP-1 using the operational model of agonism.

### Using the yeast system to investigate synthetic small-molecule GLP-1 receptor agonists

GLP-1 receptor agonism in human islets promotes insulin release (Seino, 2012); it has therefore become an increasingly attractive target for the treatment of type 2 diabetes. Several orally active, non-peptidyl agonists of the GLP-1 receptor have been described including the substituted quinoxaline, compound 2 (Knudsen *et al*., 2007) and the pyrimidine-based, BETP (also termed compound B, Sloop *et al*., 2010). These molecules have been shown to bind to the receptor in allosteric sites each having differing agonist affects on GLP-1 receptor-mediated cAMP accumulation (Knudsen *et al*., 2007; Sloop *et al*., 2010).

**Figure 6:**
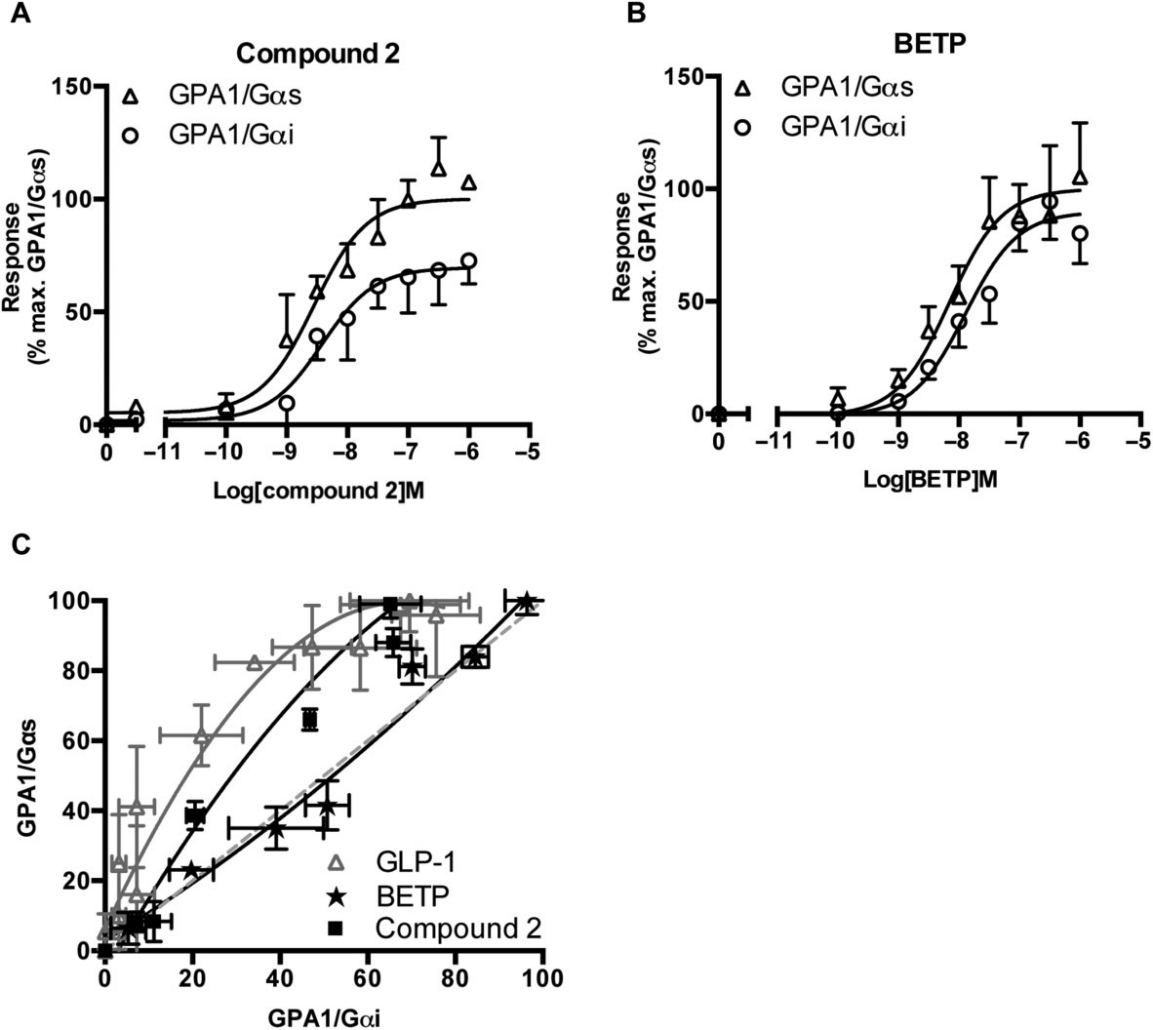
Activation of the GLP-1 receptor by non-peptide ligands. Yeast strains expressing the GLP-1 receptor containing either the GPA1/Gαs or GPA1/Gαi chimera stimulated with a range of (A), compound 2 or (B), BETP concentrations for 20 h and reporter gene activity determined. Data expressed as a percentage of the maximum response achieved through activation of GPA1/Gαs. (C) To observe pathway preferences for each ligand, the normalized responses for equivalent concentrations of ligand were used to generate an equimolar bias plot; the dashed line represents no bias. All data are mean of five independent experiments ± SEM.

Both compound 2 and BETP induced a dose-dependent increase in reporter gene activity following GLP-1 receptor stimulation (Figure 6A and B). The compound 2-induced response was significantly (*P* < 0.01, *n* = 6) influenced by the G protein chimera present displaying a reduced maximal response (Table 4) when coupled to the inhibitory pathway. In contrast, BETP agonism was not significantly (*P* > 0.05) affected by the G protein with EC_50_ values of 10 nM and 15 nM (Gαs and Gαi, respectively), this was confirmed through construction of an equimolar bias plot where the BETP data did not deviate from the no-bias line of unity (Figure 6C). These results provide a possible explanation for the observation that BETP displays partial agonism for GLP-1 receptor-mediated cAMP production in mammalian systems (Wootten *et al*., 2012) and demonstrate an intriguing insight into differences between the signalling of the two non-peptide ligands.

**Table 4:** Potency (pEC_50_) and maximal response (E_max_) values for non-peptide agonists of the GLP-1 receptor in yeast assays

Ligand	GPA1/Gαs		GPA1/Gαi	
	pEC_50_^a^	E_max_^b^	pEC_50_^a^	E_max_^b^
Compound 2	8.6 ± 0.2	100 ± 7.3	8.5 ± 0.3	67.5 ± 7.3**
BETP	8.1 ± 0.2	100 ± 5.1	7.8 ± 0.2	96.5 ± 4.3

All values determined through fitting of a three-parameter logistic equation and are mean ± SEM of five independent experimental repeats. Statistical significance was determined using Student's *t*-test.

**E_max_ GPA1/Gαi different (*P* < 0.01) from E_max_ GPA1/Gαs.

a The negative logarithm of the agonist concentration required to generate half the maximal response.

b The maximal response to the ligand expressed as a percentage of that obtained in the absence of antagonist.

### GLP-1 long-lasting mimetics display differing signalling profiles

**Figure 7:**
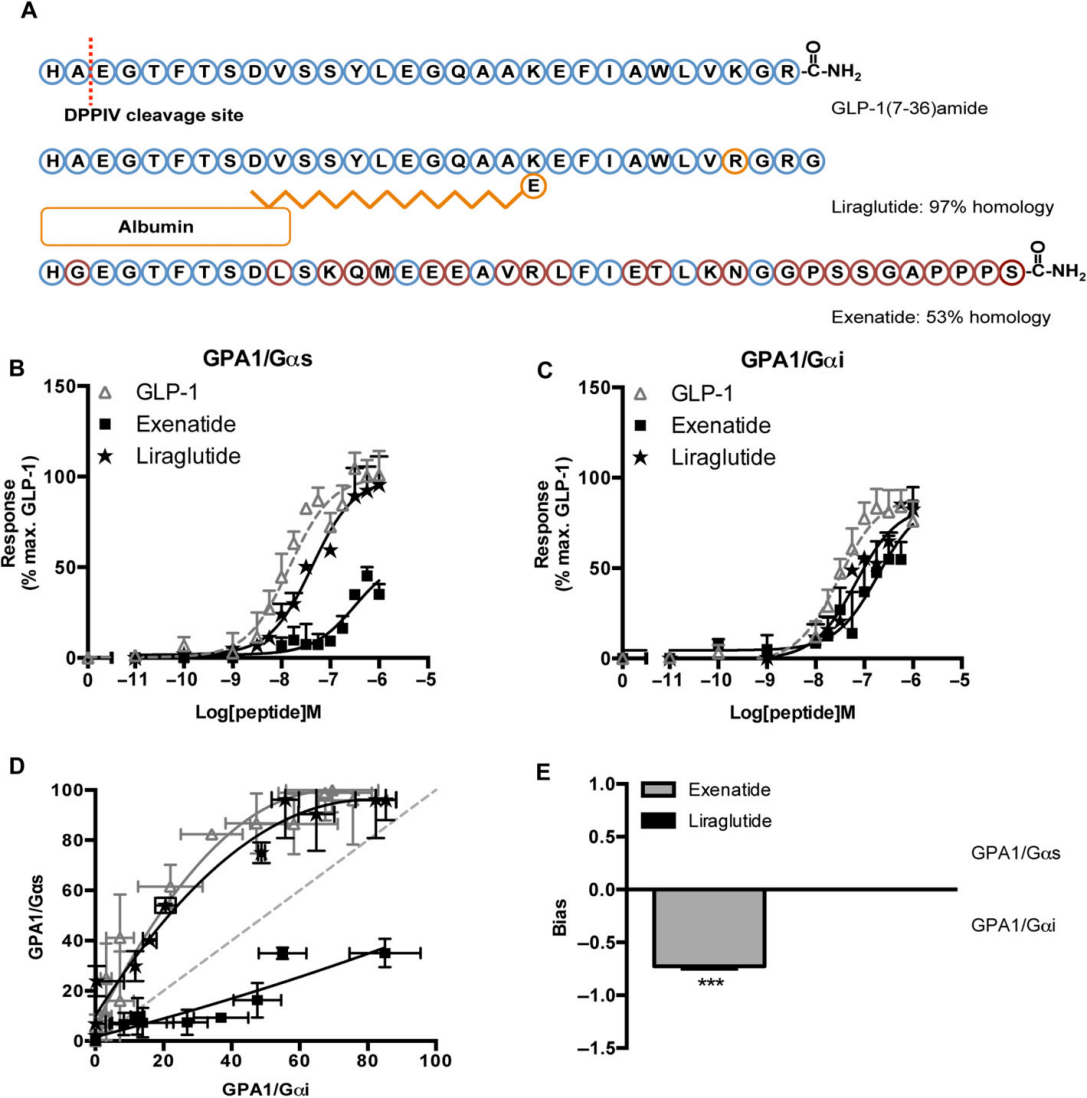
The influence of G protein subtype on GLP-1 receptor activation by peptide drugs used in the treatment of type 2 diabetes. (A) Sequence homology of GLP-1 mimetics. Blue circles are conserved throughout all three peptides, orange circles are unique to liraglutide and red denotes exenatide residues. The DPP-IV cleavage site is indicated by the dashed line. (B, C) Concentration-response curves were constructed to each of the GLP-1 receptor agonists in the indicated yeast strains. Each strain was assayed for reporter gene activity following incubation with a range of ligand concentrations for 20 h. Data are presented as the % maximal response achieved by GLP-1 at the GPA1/Gαs chimera strain. (D) Equimolar bias comparison generated by plotting the normalized responses at each G protein chimera for equivalent concentrations of ligand. Dashed line represents no bias. (E) The signalling bias was determined for each drug relative to the natural agonist using the change in log(τ/*K*_A_) ratio for the data in (B) and (C). Statistical significance was determined using one-way anova with Bonferroni's post-test with each data set compared with GLP-1 (***P < 0.001). All data are mean of 5–8 independent experiments ± SEM.

GLP-1 is rapidly broken down by peptidases in the body and a reduction in circulating peptide is observed in diabetic patients. Therefore, an alternative therapeutic approach has been to develop longer lasting peptides to prolong insulin secretion, reducing blood glucose levels in patients with Type 2 diabetes. While these injectable peptides have significant sequence homology to the natural GLP-1 agonist (liraglutide: 97% and exenatide: 53%, Figure 7A), both are relatively resistant to peptide degradation and have been approved as treatments by the US Food and Drug Administration. However, both have been associated with adverse side effects and do not exhibit the same affects in clinics (Anderson and Trujillo, 2010; Franks *et al*., 2012; Singh *et al*., 2013).

Both liraglutide and exenatide induced dose-dependent activation of the yeast-mating pathway via agonism of the GLP-1 receptor (Figure 7B and C). However, when coupled to the stimulatory GPA1/Gαs chimera, exenatide displayed a significantly (*P* < 0.001, *n* = 5) reduced EC_50_ compared with both GLP-1 and liraglutide (Table 3). Application of the operational model for partial agonism yielded log *K*_A_ (−6.4 ± 0.4 and −6.3 ± 0.4) and log τ (0.9 ± 0.3 and −0.02 ± 0.3) values for liraglutide and exenatide, respectively, further suggesting that the former acts as a partial agonist. In contrast, when coupled to the inhibitory G protein chimera, GPA1/Gαi similar potencies (Table 3) and log τ (0.5 ± 0.5 and 0.5 ± 0.4) values were obtained for both mimetics suggesting that the ligand responses are influenced by the G protein subunit present, this was further demonstrated through the generation of equimolar bias plots of the data (Figure 7D). Calculation of log(τ/*K*_A_) ratios relative to the natural agonist confirmed that liraglutide displays no change in signalling bias relative to GLP-1. However, exenatide preferentially stabilizes the GLP-1 receptor-GPA1/Gαi interaction (Figure 7E). To our knowledge, this is the first report of these differences in the pharmacology between the peptide ligands of the GLP-1 receptor and may provide valuable mechanistic insight into clinical observations.

## Discussion

Here we report the use of the *S. cerevisiae* system to isolate the individual signalling pathways, which, in human cells, are involved in GLP-1 receptor-mediated regulation of cAMP. Although a reduced potency of the natural ligand, GLP-1 was observed relative to that generated from HEK 293T cell cAMP accumulation; the yeast system is a valid platform from which to study GLP-1 receptor pharmacology with antagonism of the response faithfully reproduced. We therefore sought to use this system to investigate the molecular pharmacology of several GLP-1 receptor ligands including clinically relevant peptide mimetics and small-molecule allosteric compounds.

The advantage of yeast over other systems is the relatively null background for G protein activation and therefore the ability to observe individual activation profiles. The GLP-1 receptor, like many other GPCRs, has been documented to display coupling to numerous G proteins. Primarily, it activates the Gαs subunit to stimulate cAMP production; however, other responses including pertussis toxin-sensitive, inhibitory Gαi couplings have been observed (Montrose-Rafizadeh *et al*., 1999; Hallbrink *et al*., 2001), although these responses have been poorly characterized (Coopman *et al*., 2010). Using the yeast chimeric Gα system (Dowell and Brown, 2002), we were able to identify functional GLP-1 receptor coupling to the inhibitory G protein. The natural peptide ligand (GLP-1) promoted dose-dependent activation of the GPA1/Gαi chimera with significantly (*P* < 0.05) reduced potency (EC_50_, Table 3) and efficacy (log τ, Table 3) compared with signalling via GPA1/Gαs. However, antagonism of the response was unaffected. These data suggest that the G protein subunit present does not affect antagonist affinity and therefore any changes in agonist affinity observed are probably a result of G protein preference and not simply a consequence of the assay system. The yeast signalling platform has enabled us to investigate the potential for ligand-directed G protein signalling bias at the GLP-1 receptor.

**Figure 8:**
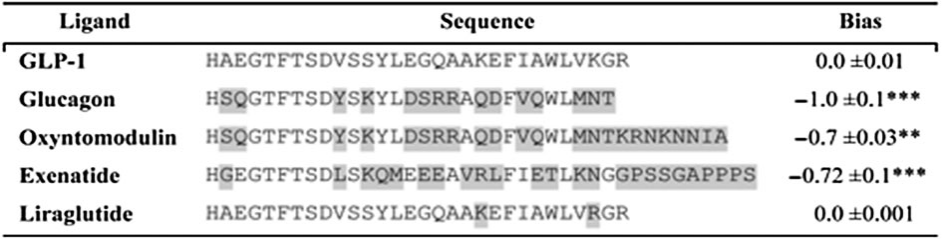
Comparison of GLP-1 receptor peptide ligands and relative bias factors. Sequences of the various peptide ligands for the GLP-1 receptor aligned to the potent, natural agonist. Amino acids differing from those in GLP-1 are highlighted in grey. The relative (to GLP-1) bias factor was quantified for each ligand as the change in log(τ/*K*_A_) ratio where a negative value indicates preference for the inhibitory, Gαi chimera. Statistical significance was determined using one-way anova with Bonferroni's post-test with each data set compared with GLP-1 (**P < 0.01, ***P < 0.001). Data are mean of 5–8 independent experiments ± SEM.

While several studies have investigated the effect of different ligands on the biasing of GLP-1 receptor signal transduction via calcium (Gαq-mediated) or ERK pathways (Koole *et al*., 2010), little has been reported for the inhibition of cAMP production. Since adverse side effects from the use of the GLP-1 mimetics could be attributed to previously unidentified off-target signalling events, we next used the yeast system to investigate ligand-induced signalling bias of other GLP-1 receptor ligands (Figure 8). The glucagon agonists, oxyntomodulin and glucagon successfully activated both the stimulatory and inhibitory G protein pathways via the GLP-1 receptor. However, both ligands displayed significant (*P* < 0.001, *n* = 5) bias towards Gαi. Similarly, the GLP-1 mimetic exenatide preferentially activated the inhibitory subunit. In contrast, only a small change in bias profile from the natural agonist was observed when liraglutide was used. Despite differences in peptide sequence primarily occurring at the C-terminus, with the exception of liraglutide, all ligands demonstrated a significant (*P* < 0.05, *n* = 5) degree of Gαi bias relative to the GLP-1. These data offer an intriguing insight into the mechanism by which peptide ligands activate family B receptors. Previous models suggest that the C-terminus is solely responsible for receptor binding to facilitate interaction of the N-terminus, which promotes activation (Hoare, 2005). Our results suggest that the C-termini of the peptides are primarily responsible for affecting the G protein preference of the activated receptor. However, given that the only peptide ligand tested that did not show a Gαi bias (liraglutide) also had the most highly conserved N-terminus, we cannot exclude the possibility that the N-terminus also plays a role in receptor signalling.

In addition to peptide ligands, the yeast system was used to investigate the signalling properties of two small-molecule allosteric compounds (compound 2 and BETP). In contrast to cAMP assays (Sloop *et al*., 2010), both ligands activated GLP-1 receptor-GPA1/Gαs with similar potencies. However, compound 2 displayed a significant reduction in maximal response when coupled to the Gαi chimera that was not observed when BETP was used. These data may provide an explanation for the observed differences in GLP-1 receptor agonism by the two allosteric compounds when cAMP production is measured (Sloop *et al*., 2010).

Multiple glucagon-related receptors have been implicated in glycaemic control, all of which couple to several G protein subunits. The ability of ligands to differentially activate specific G protein pathways following receptor stimulation has been a noted property for GPCRs for many years (Kenakin, 1995). Differential activation of these pathways including receptor internalization, Ca^2+^ signalling and cAMP regulation by ligands, results in distinct physiological responses. The yeast system enables investigation of individual receptor-Gα subunit interactions and therefore provides a simple, robust assay to identify compounds that affect particular pathways. Here we have reported the functional expression of two glucagon-related receptors, which act in opposition to regulate blood glucose levels. Interestingly, a number of naturally occurring ligands (glucagon and oxyntomodulin) have been documented to activate both the glucagon receptors and the GLP-1 receptor (Jorgensen *et al*., 2007). However, each of the different ligand–receptor combinations displays a distinct G protein bias profile. It has been suggested that it may be beneficial to generate so-called hybrid peptides, which could modulate the activity of both receptors (in different ways) simultaneously (Cho *et al*., 2012). For example, a peptide that functioned as an agonist for GLP-1 receptor-Gαs while antagonizing the glucagon receptor-Gαs could have enormous therapeutic potential. Selecting compounds that differentially regulate the G protein pathway activated by each receptor could have a similar effect. For example, a molecule, which activates GLP-1 receptor-Gαs and also glucagon receptor -Gαi, would appear to antagonize the latter's cAMP response. The observations in this manuscript that the simple yeast-screening platform can not only be used to screen for receptor activation but also to detect ligand-directed G protein-signalling bias for these receptors demonstrates that it has the potential to be used in the rapid identification of such compounds.

The GLP-1 receptor not only regulates the release of insulin, but it has also been implicated in a host of other clinical contexts including cardioprotective and satiety effects (Pabreja *et al*., 2014). It is thought that these effects are mediated from different tissues. Indeed, the GLP-1 receptor has been isolated from multiple tissues including lung, brain, stomach and heart (reviewed Cho *et al*., 2012). Several reports have noted that GLP-1 receptors display tissue-specific pharmacology (reviewed Pabreja *et al*., 2014) leading to the suggestion of a second GLP-1 receptor subtype; however, this has not been identified. Given our observations of ligand-directed G protein signalling bias, it is tempting to speculate that these multiple receptor subtypes could result from tissue-specific GLP-1 receptor-G protein couplings. For example, activation of the GLP-1 receptor inhibits the release of gastric acid release thereby delaying gastric emptying. The mechanism by which this occurs has not been fully elucidated; however, there is some suggestion that it is pertussis toxin-sensitive Gαi coupling (Schepp *et al*., 1992). A systematic review of cell type-specific GLP-1 receptor signal transduction has not, to date, been undertaken in particular with reference to responses mediated via different G protein subunits, which would be needed to provide clarity. Given the wide range of off-target side effects reported from the use of the clinically prescribed drugs and our data indicating their distinct G protein bias profiles, not only Gαs pathways should be considered but also those responses mediated through Gαi in each tissue.

The data we have described demonstrate the powerful ability of the yeast assay to observe, for the first time, system-independent, pharmacological properties of potential therapeutic agents. Both the glucagon receptor and the GLP-1 receptor are members of the secretin (family B, class 2) receptor family, which contains 15 GPCRs. These GPCRs typically bind peptide ligands that range in size from 27 to 84 amino acids. Consequently, for many years, there was reluctance from researchers to explore these receptors in yeast due to the presence of the yeast cell wall. It was argued that peptides would be restricted in their ability to permeate the cell wall so preventing activation of the GPCR. We have previously reported the expression of two corticotropin-releasing factor receptor subtypes in yeast (Ladds *et al*., 2003; 2005a), while the functional co-expression of the calcitonin receptor-like receptor with various receptor activity modifying proteins (RAMPs) has also been reported (Miret *et al*., 2002). In all cases, as with the glucagon receptor and GLP-1 receptor, the pharmacology of the receptors is faithfully transferred to the yeast all-be-it with a reduced affinity for the ligands.

Extension of the technology to include many GPCRs or other proteins, which may interact with the receptor *in situ* (e.g. RAMPs and regulator of G protein signalling proteins), would enable the observation of potential physiological changes in receptor pharmacology. Additionally, the relative simplicity of receptor expression within the yeast system would allow the introduction of patient-specific mutations to quantify their effects of ligand signalling potentially leading to efficient screening of personalized drugs.

## Abbreviations

BETP: 4-(3-benzyloxyphenyl)-2-ethylsufinyl-6-(trifluoromethyl)pyrimidine
compound 2: 6,7-dichloro-2-methylsulfonyl-3-N-tert-butylaminoquinoxaline
DPP-IV: dipeptidyl peptidase-IV
FDGlu,: flurorescein-Di-β-D-glucopyranoside
GLP-1: glucagon-like peptide-1; GPCR, G-protein coupled receptor

## References

[b1] Alexander SPH, Benson HE, Faccenda E, Pawson AJ, Sharman JL, Spedding M, Peters JA, Harmar AJ, CGTP Collaborators (2013). The Concise Guide to PHARMACOLOGY 2013/14: G protein-coupled receptors. Br J Pharmacol.

[b2] Anderson SL, Trujillo JM (2010). Association of pancreatitis with glucagon-like peptide-1 agonist use. Ann Pharmacother.

[b3] Baggio LL, Drucker DJ (2007). Biology of Incretins: GLP-1 and GIP. Gastroenterology.

[b4] Bertheleme N, Singh S, Dowell SJ, Hubbard J, Byrne B (2013). Loss of constitutive activity is correlated with increased thermostability of the human adenosine A_2A_ receptor 1. Br J Pharmacol.

[b5] Black JW, Leff P (1983). Operational models of pharmacological agonism. Proc R Soc Lond B Biol Sci.

[b6] Bond M, Croft W, Tyson R, Bretschneider T, Davey J, Ladds G (2013). Quantitative analysis of human ras localization and function in the fission yeast *Schizosaccharomyces pombe*. Yeast.

[b7] Brown AJ, Dyos SL, Whiteway MS, White JH, Watson MAE, Marzioch M (2000). Functional coupling of mammalian receptors to the yeast mating pathway using novel yeast/mammalian G protein α-subunit chimeras. Yeast.

[b8] Brown AJ, Goldsworthy SM, Barnes AA, Eilert MM, Tcheang L, Daniels D (2003). The orphan G protein-coupled receptors GPR41 and GPR43 are activated by propionate and other short chain carboxylic acids. J Biol Chem.

[b9] Butler AE, Campbell-Thompson M, Gurlo T, Dawson DW, Atkinson M, Butler PC (2013). Marked expansion of exocrine and endocrine pancreas with incretin therapy in humans with increased exocrine pancreas dysplasia and the potential for glucagon-producing neuroendocrine tumors. Diabetes.

[b10] Cho YM, Merchant CE, Kieffer TJ (2012). Targeting the glucagon receptor family for diabetes and obesity therapy. Pharmacol Ther.

[b11] Coopman K, Huang Y, Johnston N, Bradley SJ, Wilkinson GF, Willars GB (2010). Comparative effects of the endogenous agonist glucagon-like peptide-1 (GLP-1)-(7-36) amide and the small-molecule ago-allosteric agent ‘compound 2’ at the GLP-1 receptor. J Pharmacol Exp Ther.

[b12] Dohlman HG, Apaniesk D, Chen Y, Song J, Nusskern D (1995). Inhibition of G-protein signaling by dominant gain-of-function mutations in Sst2p, a pheromone desensitization factor in Saccharomyces cerevisiae. Mol Cell Biol.

[b13] Dowell SJ, Brown AJ (2002). Yeast assays for G-protein-coupled receptors. Receptors Channels.

[b14] Dowell SJ, Brown AJ (2009). Yeast assays for G protein-coupled receptors. G Protein-Coupled Receptors in Drug Discovery.

[b15] Figueroa KW, Griffin MT, Ehlert FJ (2009). Selectivity of agonists for the active state of M1 to M4 muscarinic receptor subtypes. J Pharmacol Exp Ther.

[b16] Franks AS, Lee PH, George CM (2012). Pancreatitis: a potential complication of liraglutide?. Ann Pharmacother.

[b17] Gaddum JH, Hameed KA, Hathway DE, Stephens FF (1955). Quantitative studies of antagonists for 5-hydroxytryptamine. Q J Exp Physiol Cogn Med Sci.

[b18] Gietz RD, Schiestl RH (2007). High-efficiency yeast transformation using the LiAc/SS carrier DNA/PEG method. Nat Protoc.

[b19] Göke R, Fehmann HC, Linn T, Schmidt H, Krause M, Eng J (1993). Exendin-4 is a high potency agonist and truncated exendin-(9-39)-amide an antagonist at the glucagon-like peptide 1-(7-36)-amide receptor of insulin-secreting beta-cells. J Biol Chem.

[b20] Hallbrink M, Holmqvist T, Olsson M, Ostenson CG, Efendic S, Langel U (2001). Different domains in the third intracellular loop of the GLP-1 receptor are responsible for Galpha(s) and Galpha(i)/Galpha(o) activation. Biochim Biophys Acta.

[b21] Hoare SR (2005). Mechanisms of peptide and nonpeptide ligand binding to class B G-protein-coupled receptors. Drug Discov Today.

[b22] Holst JJ, Seino Y (2009). GLP-1 receptor agonists: targeting both hyperglycaemia and disease processes in diabetes. Diabetes Res Clin Pract.

[b23] Jorgensen R, Kubale V, Vrecl M, Schwartz TW, Elling CE (2007). Oxyntomodulin differentially affects glucagon-like peptide-1 receptor β-arrestin recruitment and signaling through Gα. J Pharmacol Exp Ther.

[b24] Kenakin TP (1995). Agonist-receptor efficacy II: agonist trafficking of receptor signals. Trends Pharmacol Sci.

[b25] Knudsen LB, Kiel D, Teng M, Behrens C, Bhumralkar D, Kodra JT (2007). Small-molecule agonists for the glucagon-like peptide 1 receptor. Proc Natl Acad Sci U S A.

[b26] Koole C, Wootten D, Simms J, Valant C, Sridhar R, Woodman OL (2010). Allosteric ligands of the glucagon-like peptide 1 receptor (GLP-1R) differentially modulate endogenous and exogenous peptide responses in a pathway-selective manner: implications for drug screening. Mol Pharmacol.

[b27] Ladds G, Davey J (2000). Sxa2 is a serine carboxypeptidase that degrades extracellular P-factor in the fission yeast *Schizosaccharomyces pombe*. Mol Microbiol.

[b28] Ladds G, Davis K, Hillhouse EW, Davey J (2003). Modified yeast cells to investigate the coupling of G protein-coupled receptors to specific G proteins. Mol Microbiol.

[b29] Ladds G, Goddard A, Davey J (2005a). Functional analysis of heterologous GPCR signalling pathways in yeast. Trends Biotechnol.

[b30] Ladds G, Davis K, Das A, Davey J (2005b). A constitutively active GPCR retains its G protein specificity and the ability to form dimers. Mol Microbiol.

[b31] McIntyre N, Holdsworth CD, Turner DS (1964). New interpretation of oral glucose tolerance. Lancet.

[b32] Mentlein R, Gallwitz B, Schmidt WE (1993). Dipeptidyl-peptidase IV hydrolyses gastric inhibitory polypeptide, glucagon-like peptide-1(7–36)amide, peptide histidine methionine and is responsible for their degradation in human serum. Eur J Biochem.

[b33] Miret JJ, Rakhilina L, Silverman L, Oehlen B (2002). Functional expression of heteromeric calcitonin gene-related peptide and adrenomedullin receptors in yeast. J Biol Chem.

[b34] Montrose-Rafizadeh C, Avdonin P, Garant MJ, Rodgers BD, Kole S, Yang H (1999). Pancreatic glucagon-like peptide-1 receptor couples to multiple G proteins and activates mitogen-activated protein kinase pathways in Chinese hamster ovary cells. Endocrinology.

[b35] Nathan DM, Buse JB, Davidson MB, Ferrannini E, Holman RR, Sherwin R (2009). Medical management of hyperglycemia in type 2 diabetes: a consensus algorithm for the initiation and adjustment of therapy: a consensus statement of the American Diabetes Association and the European Association for the Study of Diabetes. Diabetes Care.

[b36] Nauck MA (2011). Incretin-based therapies for type 2 diabetes mellitus: properties, functions, and clinical implications. Am J Med.

[b37] Pabreja K, Mohd MA, Koole C, Wootten D, Furness SGB (2014). Molecular mechanisms underlying physiological and receptor pleiotropic effects mediated by GLP-1R activation. Br J Pharmacol.

[b38] Poyner DR, Andrew DP, Brown D, Bose C, Hanley MR (1992). Pharmacological characterization of a receptor for calcitonin gene-related peptide on rat, L6 myocytes. Br J Pharmacol.

[b39] Quddusi S, Vahl TP, Hanson K, Prigeon RL, D'Alessio DA (2003). Differential effects of acute and extended infusions of glucagon-like peptide-1 on first- and second-phase insulin secretion in diabetic and nondiabetic humans. Diabetes Care.

[b40] Rajagopal S, Ahn S, Rominger DH, Gowen-MacDonald W, Lam CM, DeWire SM (2011). Quantifying ligand bias at seven-transmembrane receptors. Mol Pharmacol.

[b41] Schepp W, Schmidtler J, Dehne K, Schusdziarra V, Classen M (1992). Pertussis toxin-sensitive and pertussis toxin-insensitive inhibition of parietal cell response to GLP-1 and histamine. Am J Physiol Gastrointest Liver Physiol.

[b42] Schepp W, Dehne K, Riedel T, Schmidtler J, Schaffer K, Classen M (1996). Oxyntomodulin: a cAMP-dependent stimulus of rat parietal cell function via the receptor for glucagon-like peptide-1 (7-36) NH2. Digestion.

[b43] Scott DA, Boye KS, Timlin L, Clark JF, Best JH (2013). A network meta-analysis to compare glycaemic control in patients with type 2 diabetes treated with exenatide once weekly or liraglutide once daily in comparison with insulin glargine, exenatide twice daily or placebo. Diabetes Obes Metab.

[b44] Seino S (2012). Cell signalling in insulin secretion: the molecular targets of ATP, cAMP and sulfonylurea. Diabetologia.

[b45] Singh S, Chang H-Y, Richards TM, Weiner JP, Clark JM, Degal JB (2013). Glucagon-like peptide-1-based therapies and risk of hospitalization for acute pancreatitis in type 2 diabetes mellitus: a population-based matched case-control study. JAMA Intern Med.

[b46] Sloop KW, Willard FS, Brenner MB, Ficorilli J, Valasek K, Showalter AD (2010). Novel small molecule glucagon-like peptide-1 receptor agonist stimulates insulin secretion in rodents and from human islets. Diabetes.

[b47] Stewart GD, Valant C, Dowell SJ, Mijaljica D, Devenish RJ, Scammells PJ (2009). Determination of adenosine A_1_ receptor agonist and antagonist pharmacology using *Saccharomyces cerevisiae*: implications for ligand screening and functional selectivity. J Pharmacol Exp Ther.

[b48] Wootten D, Savage EE, Valant C, May LT, Sloop KW, Ficorilli J (2012). Allosteric modulation of endogenous metabolites as an avenue for drug discovery. Mol Pharmacol.

[b49] Wynne K, Park AJ, Small CJ, Patterson M, Ellis SM, Murphy KG (2005). Subcutaneous oxyntomodulin reduces body weight in overweight and obese subjects a double-blind, randomized, controlled trial. Diabetes.

